# Rice plants have three homologs of glutathione synthetase genes, one of which, *OsGS2*, codes for hydroxymethyl‐glutathione synthetase

**DOI:** 10.1002/pld3.119

**Published:** 2019-02-18

**Authors:** Shinichi Yamazaki, Kumiko Ochiai, Toru Matoh

**Affiliations:** ^1^ Graduate School of Agriculture Kyoto University Kyoto Japan

**Keywords:** glutathione, glutathione synthetase, hmGSH, rice

## Abstract

Glutathione is a ubiquitous thiol tripeptide in land plants, and glutathione‐like tripeptides can also be found in some plant species. Rice (*Oryza sativa*) plants synthesize hydroxymethyl‐glutathione, in which the terminal glycine residue of glutathione is replaced by a serine residue; however, the biosynthetic pathway of hydroxymethyl‐glutathione has not been identified. We isolated three rice glutathione synthetase homologs, designated OsGS1, OsGS2, and OsGS3, and found that knockdown of *OsGS2* via RNA interference markedly decreased hydroxymethyl‐glutathione concentration in rice plants. The in vitro enzyme assay, using purified recombinant protein, demonstrated that OsGS2 catalyzed the synthesis of hydroxymethyl‐glutathione from γ‐glutamylcysteine (γEC) and L‐serine in an ATP‐dependent manner. OsGS2 could also utilize glycine as a cosubstrate with γEC, but the enzyme‐substrate affinity for L‐serine was tenfold higher than that for glycine. These results indicate that *OsGS2* codes for hydroxymethyl‐glutathione synthetase.

## 1 INTRODUCTION

The tripeptide glutathione (GSH; γGlu‐Cys‐Gly) is synthesized from its constituent amino acids through two steps of ATP‐dependent reactions, catalyzed by γ‐glutamylcysteine synthetase (γECS or GSH1) and glutathione synthetase (GS or GSH2), and it has multiple functions in cellular metabolisms. In *Arabidopsis thaliana*, knockout of either AtGSH1 or AtGSH2 is lethal to plants (Cairns, Pasternak, Wachter, Cobbett, & Meyer, 2006; Pasternak et al., 2008), while a decrease in GSH levels due to a mutation in *AtGSH1* gene results in a failure to develop a meristem (Vernoux et al., 2000), demonstrating that GSH is essential for plant development. In addition, GSH maintains cellular redox homeostasis and acts as a scavenger of reactive oxygen species induced by environmental stresses (Hernández et al., 2015; Jozefczak et al., 2014). Conjugate formation of GSH with xenobiotics and metabolites catalyzed by glutathione *S*‐transferases (GSTs) is also an important step in detoxification and secondary metabolism in plants (Dixon & Edwards, 2010). In *Arabidopsis*, tolerance to toxic metals, such as cadmium (Cd) and arsenic (As), is also mediated by GSH and phytochelatins (PCs), the latter being polymerized peptides with the structure of (γGlu‐Cys)_n_‐Gly, synthesized from GSH in response to heavy metals and metalloids (Cobbett, 2000; Ha et al., 1999; Howden, Goldsbrough, Andersen, & Cobbett, 1995; Schmöger, Oven, & Grill, 2000; Verbruggen, Hermans, & Schat, 2009).

In some plant species, other thiol tripeptides are present with a structure similar to that of GSH, in which the glycine residue is replaced by other amino acids. Leguminous plants produce homo‐glutathione (hGSH; γGlu‐Cys‐βAla) (Klapheck, 1988); some poaceous plants, including wheat (*Triticum aestivum*), barley (*Hordeum vulgare*), and rice (*Oryza sativa*), synthesize hydroxymethyl‐glutathione (hmGSH; γGlu‐Cys‐Ser) (Klapheck, Chrost, Starke, & Zimmermann, 1992), and maize (*Zea mays*) contains γGlu‐Cys‐Glu (γECE) (Meuwly, Thibault, & Rauser, 1993). Among those GSH‐like tripeptides, hGSH is known to be synthesized from γ‐glutamylcysteine (γEC) and β‐alanine (βAla) in a reaction catalyzed by homo‐glutathione synthetase (hGS), which is homologous to the GS enzyme (Frendo et al., 2001). However, the biosynthetic pathways of other GSH‐like tripeptides have not been identified. Since recombinant GS proteins from wheat and maize, namely, TaGS1, TaGS2, and ZmGS, could not synthesize hmGSH and γECE from γEC and the corresponding amino acids, it has been suggested that those GSH‐like tripeptides arose from postsynthetic modification of GSH (Skipsey, Davis, & Edwards, 2005). Rice plants also accumulate high concentrations of hmGSH under Cd stress (Klapheck, Fliengner, & Zimmer, 1994), but the physiological roles of the thiol tripeptide have not been clarified.

In this study on rice, we identified genes involved in GSH synthesis and the enzyme that is responsible for hmGSH synthesis.

## 2 MATERIALS AND METHODS

### 2.1 Plant material and growth conditions

A *japonica* rice cultivar “Nipponbare” and transgenic T_1_ RNAi plants generated from this cultivar were used in this study. Seeds were soaked for 3 days at 30°C in distilled water containing 0.3% (w/v) fungicide (Trifmine; Nippon Soda Co., Ltd.). Seeds were then sown on plastic meshes floating on a culture solution. Seedlings were grown hydroponically in an incubator at 30°C and 80% relative humidity, under a 12‐hr light period. The culture solution contained 1 mmol/L (NH_4_)_2_SO_4_, 0.5 mmol/L KCl, 0.25 mmol/L KH_2_PO_4_, 0.5 mmol/L CaCl_2_, and 0.5 mmol/L MgCl_2_ for macronutrients and 89.5 μmol/L EDTA‐FeNa, 46.3 μmol/L H_3_BO_3_, 9.1 μmol/L MnCl_2_·4H_2_O, 0.3 μmol/L CuSO_4_·5H_2_O, 0.8 μmol/L ZnSO_4_·7H_2_O, and 0.1 μmol/L (NH_4_)_6_Mo_7_O_24_·4H_2_O for micronutrients (Hoagland & Arnon, 1950). In Cd stress condition, CdSO_4_ was added into the culture solution at 10 μmol/L. At each sampling date, roots and basal stems were washed with distilled water for 30 s and gently blotted on paper towels.

### 2.2 Homology search and sequence analysis of GS genes

Through the BLAST search of the Rice Annotation Project database (RAP‐DB), using the amino acid sequence of *Arabidopsis* GS (AtGS) as a query, three rice *GS*‐like genes were hit, *Os11g0642800*,* Os12g0263000*, and *Os12g0528400*, and we designated them as *OsGS1*,* OsGS2*, and *OsGS3*, respectively. The transcript sequence of *OsGS1* (accession no. AK068792) had a full‐length ORF sequence, but those of the others (*OsGS2*, AK099555; *OsGS3*, AK103004 and AK058922) contained only partial coding regions. Thus, we repeated the BLAST search of the National Center for Biotechnology Information (NCBI), using the transcript sequences of AK099555 for *OsGS2* and AK103004 for *OsGS3* as queries. Candidate transcript sequences were found (*OsGS2*, EU267952; *OsGS3*, XM_015762575). The *OsGS2* transcript sequence (EU267952) was a full‐length *GS*‐like transcript derived from the *japonica* cultivar “Ilpumbyeo”, and its DNA sequence in the exon regions was identical to the corresponding genome regions of “Nipponbare”. The *OsGS3* sequence (XM_015762575) was a *GS*‐like transcript predicted by computational analysis.

To confirm those sequences, we performed sequencing analysis of PCR‐amplified product using “Nipponbare” cDNA as template. For the amplification, the following primer sets were used: GS2.RNAi.F and GS2.seqR for the *OsGS2* fragment and GS3.seqF and GS3.RNAi.R for the *OsGS3* fragment (Table S1). PCR amplification was carried out using Blend Taq DNA polymerase (TOYOBO). The amplified products were purified using a Mag Extractor (TOYOBO) and then sequenced under contract (FASMAC). For the sequencing analysis, the primers mentioned above and the following ones were used: GS2.ex‐F and GS2.ex‐R for the *OsGS2* fragment and GS3.ex‐R for the *OsGS3* fragment (Table S1). The transcript sequences obtained from the analysis were identical to the sequences, EU267952 and XM_015762575, and *OsGS3* sequence data were submitted to the DNA Data Bank of Japan (LC385880).

### 2.3 Phylogenetic tree

The phylogenetic analysis of *GS* and *hGS* genes from different species of higher plants was performed using the neighbor‐joining method of the Clustal W 2.1 program (Larkin et al., 2007). Abbreviation and GenBank accession numbers are as follows: AtGS (*Arabidopsis thaliana* GS, AJ243813), BjGS (*Brassica juncea* GS, Y10984), Gm‐hGS (*Glycine max* hGS, AJ272035), LjGS (*Lotus japonicus* GS, AF279703), Lj‐hGS (*L. japonicus* hGS, AY219337), MtGS (*Medicago truncatula* GS, AF194421), Mt‐hGS (*M. truncatula* hGS, AF075700), PsGS (*Pisum sativum* GS, AF231137), Ps‐hGS (*P. sativum* hGS, AF258319), ZmGS (*Zea mays* GS, AJ579383), TaGS1 (*Triticum aestivum* GS1, AJ579380), TaGS2 (*T. aestivum* GS2, AJ579381), TaGS3 (*T. aestivum* GS3, AJ579382), TaGS4 (*T. aestivum* predicted GS mRNA, AK455907), HvGS1 (*Hordeum vulgare* predicted GS mRNA, AK354495) and HvGS2 (*H. vulgare* predicted GS mRNA, AK364878).

### 2.4 Gene expression analysis

For the analysis of *OsGS* expression levels in WT plants (Figure 2), total RNA was extracted from shoots or roots of three bulked plants using the Plant Total RNA Extraction MiniPrep System (VIOGENE). For the analysis of transgenic RNAi plants (Figure 3), total RNA was extracted from the shoots or roots of individual plants. Genomic DNA in the extract was digested with recombinant DNase I (TaKaRa Bio). Single‐stranded cDNA was synthesized using an oligo (dT)_20_ primer and ReverTra Ace Reverse Transcriptase (TOYOBO). Gene expression levels were quantified by RT‐qPCR performed with a THUNDERBIRD SYBR qPCR Mix (TOYOBO). The geometric mean of the expression levels of the *ubiquitin* and *Actin1* genes were used as the internal standards. The sequences of primers used in the experiment are shown in Table S1.

### 2.5 Transformation of rice to generate RNAi plants

*OsGS* RNAi rice plants were generated by *Agrobacterium*‐mediated transformation (Toki et al., 2006). Segments of 306 bp, 289 bp, or 290 bp sequences specific to *OsGS1*,* OsGS2*, or *OsGS3*, respectively, were used as the RNAi trigger. Alignment of RNAi trigger sequences is shown in Figure S1, and their identity matrix of them were in the range of 39.61%–53.82% (Table S2). The trigger sequence was amplified by PCR using Prime Star DNA polymerase (TaKaRa) and the appropriate primer set, GS1.RNAi.F and GS1.RNAi.R, GS2.RNAi.F and GS2.RNAi.R, or GS3.RNAi.F and GS3.RNAi.R (Table S1). The forward primers contained a CACC sequence at the 5′ end for TOPO cloning. The amplified fragment was cloned into the Gateway pENTR/D‐TOPO cloning vector (Invitrogen).

The fragment was subsequently inserted into the pANDA destination vector, which was under the control of a constitutive maize *ubiquitin1* promoter (Miki & Shimamoto, 2004). The RNAi construct was introduced into *Agrobacterium tumefaciens* strain EHA105 (Hood, Gelvin, Melchers, & Hoekema, 1993), which was used to transform rice cv. Nipponbare, as described by Toki et al. (2006). In the transgenic plant experiment, after separating the shoots and roots at the time of sampling, residual pieces of individual RNAi T_1_ plants were used to extract genomic DNA. The genotype of individual plants was identified by PCR, using Blend Taq DNA polymerase (TOYOBO) and the primer sets, Gus‐linker.R and the corresponding RNAi.R (Table S1).

### 2.6 Preparation and purification of recombinant proteins

Full‐length ORF sequences of *OsGS1* and *OsGS2* were amplified by PCR using Prime Star DNA polymerase (TaKaRa Bio), “Nipponbare” cDNA as the template, and the primer sets, GS1.proF and GS1.proR, or GS2.proF and GS2.proR (Table S1). The *OsGS3* full‐length sequence was amplified by nested PCR using the following primer sets: step 1, GS3.seqF and GS3.proR; step 2, GS3.proF and GS3.proR. The reverse primers contained a recognition sequence for SbfI (GGACGTCC) at the 5′ end. The amplified fragment was subcloned into the pMAL‐c5X vector (New England Biolabs) following the manufacturer's instruction. The sequences of subcloned fragments were confirmed by sequencing analysis, using the primers shown in Table S1. The recombinant maltose‐binding protein (MBP)‐fusion protein was expressed in the *Escherichia coli* strain NEB Express (ER2523; New England Biolabs) and purified by affinity chromatography using amylose resin (Kellermann & Ferenci, 1982). Protein purity was confirmed by SDS‐PAGE (Figure S2).

### 2.7 GS enzyme assay

The reaction mixture (100 μl volume) contained 100 mmol/L Tris‐HCl buffer (pH 8.0), 50 mmol/L KCl, 20 mmol/L MgCl_2_, 1 mmol/L dithiothreitol (DTT), 0.1 mg/ml BSA, 2 mmol/L ATP, 1 mmol/L γEC, and 4 mmol/L amino acid (Gly, L‐Ser, or L‐Glu). The solution was pre‐incubated at 30°C for 15 min, and the reaction was started by the addition of the purified recombinant protein. MBP‐OsGS1 or MBP‐OsGS2 was added at 10 μg/ml, while MBP‐OsGS3 was added at 0.5 μg/ml because it had a higher specific activity than other two OsGS proteins. The reaction was carried out at 30°C for 30 min and was stopped by the addition of trifluoroacetic acid (TFA) at a final concentration of 1% (v/v).

In the kinetic analyses, Gly or L‐Ser was added at 0.2, 0.4, 0.6, 1, 2, 4, or 10 mmol/L in the reaction mixture (100 μl volume). At each of 5, 10, 15, and 20 min from the start of reaction, a 20 μl aliquot of the assay solution was collected and mixed with 5 μl of 5% (v/v) TFA to stop the enzyme reaction. The assay solution aliquots were stored at −20°C until analysis was carried out.

### 2.8 Quantification of thiol peptides

The concentrations of thiol peptides in rice plants were quantified as described by Sneller et al. (2000), with some modifications. Shoot samples were freeze‐dried and pulverized using a ball mill. Thiol peptides were extracted from 10 mg of the sample powder by adding 250 μl of 0.1% (v/v) TFA containing 6.3 mmol/L diethylenetriaminepentaacetic acid (DTPA). After centrifugation at 4°C, 100 μl of the supernatant was collected and mixed with 180 μl of 200 mmol/L HEPES buffer (pH 8.2) containing 6.3 mmol/L DTPA. To this solution, 10 μl of 20 mmol/L DTT was added to reduce the thiols. After standing for 30 min at room temperature, 10 μl of 50 mmol/L monobromobimane (mBBr), dissolved in dimethyl sulfoxide, was added, and a derivatization reaction was performed for 1 hr at room temperature in the dark. The reaction was stopped by the addition of 120 μl of 1 mol/L methanesulfonic acid. The derivatized sample solution was kept on ice in the dark until analysis was carried out.

For the quantification of the thiol peptides synthesized by the recombinant proteins, 15 μl of the reaction product solution was mixed with 135 μl of 0.1% (v/v) TFA containing 6.3 mmol/L DTPA. After centrifugation at 4°C, 100 μl solution was collected and derivatized with mBBr as described above, except that no DTT was added.

Following filteration with a Cosmospin Filter G (pore size 0.2 μm; NACALAI TESQUE), a 20 μl aliquot of the filtrate of the derivatized sample was injected into an HPLC system (LC‐10AS; Shimadzu) equipped with a Hypersil ODS C18 column (5 μm; 12 nm, 4.6 × 250 mm; Thermo Fisher Scientific Inc.). Elution was performed at a flow rate of 0.5 ml/min with a linear gradient of methanol and water for 60 min for analysis of plant samples or 30 min for analysis of enzyme reaction products. Eluent A [10% (v/v) methanol and 90% water containing 0.05% (v/v) TFA] and eluent B (80% methanol and 20% water containing 0.05% TFA) were used in a linear gradient elution with the following time program for plant sample: at 0 min, eluent B was 28%; 15 min, 30%; 30 min, 40%; 35 min, 45%; 40 min, 58%; 45 min, 75%; 48 min, 90%; 49 min, 100%; 55 min, 28%; and 60 min, 28%. The time program for enzyme reaction product was as follows: at 0 min, eluent B was 28%; 10 min, 30%; 12 min, 40%; 16 min, 70%; 19 min, 100%; 25 min, 28%; and 30 min, 28%. The elutant was monitored using a fluorescence detector (RF‐10A_XL_; Shimadzu) with the excitation wavelength set at 380 nm and the emission wavelength at 470 nm.

Authentic reagents such as GSH (NACALAI TESQUE), hmGSH, γEC, and γECE (HiPep Laboratories) were used to identify the elution peaks of each thiol peptide. Thiol peptides were quantified by the GSH standard.

### 2.9 Statistical analysis

The data were expressed as mean ± *SE* from at least three biological replicates for each experiment. Statistical significance was determined using Dunnett's test for multiple comparisons with the control. Non‐linear regression analysis was used to determine the equation of the curve‐of‐best‐fit for the Michaelis‐Menten equation.

### 2.10 Accession number

The nucleotide sequence reported in this paper has been submitted to the DNA Data Bank of Japan with the accession number LC385880.

## 3 RESULTS

### 3.1 Isolation and expression levels of rice glutathione synthetase homolog genes

**Figure 1:**
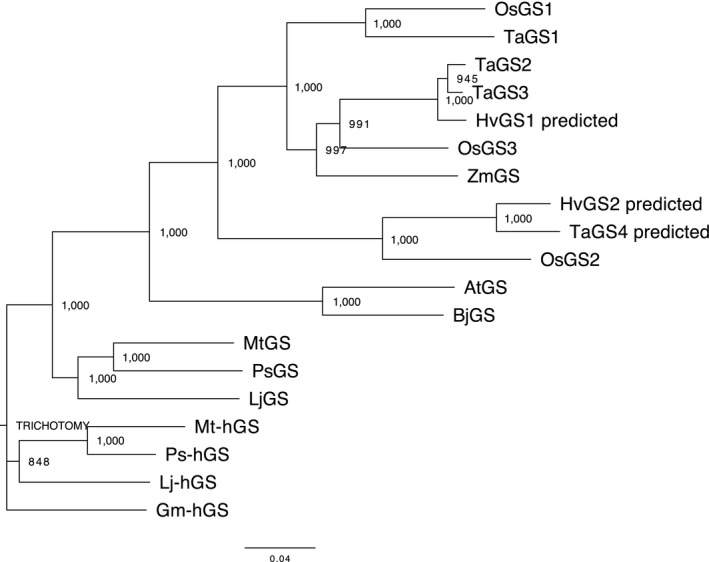
Phylogenetic tree of plant GS and hGS genes. The tree was calculated using the neighbor‐joining method of the Clustal W 2.1 program. The numbers correspond to frequencies of 1,000 bootstraps. The bar represents 0.04 substitutions per site. Abbreviation and GenBank accession numbers are as follows: AtGS (*Arabidopsis thaliana* GS, AJ243813), BjGS (*Brassica juncea* GS, Y10984), Gm‐hGS (*Glycine max* hGS, AJ272035), LjGS (*Lotus japonicus* GS, AF279703), Lj‐hGS (*L. japonicus* hGS, AY219337), MtGS (*Medicago truncatula* GS, AF194421), Mt‐hGS (*M. truncatula* hGS, AF075700), PsGS (*Pisum sativum* GS, AF231137), Ps‐hGS (*P. sativum* hGS, AF258319), ZmGS (*Zea mays* GS, AJ579383), TaGS1 (*Triticum aestivum* GS1, AJ579380), TaGS2 (*T. aestivum* GS2, AJ579381), TaGS3 (*T. aestivum* GS3, AJ579382), TaGS4 (*T. aestivum* predicted GS mRNA, AK455907), HvGS1 (*Hordeum vulgare* predicted GS mRNA, AK354495) and HvGS2 (*H. vulgare* predicted GS mRNA, AK364878)

In a homology search conducted with the amino acid sequence of *Arabidopsis* glutathione synthetase (AtGS) as a query, we found three homologous genes in the rice genome, *Os11g0642800*,* Os12g0263000*, and *Os12g0528400*, and designated them as *OsGS1*,* OsGS2*, and *OsGS3*, respectively. By sequencing analysis using “Nipponbare” cDNA, the transcript sequence of each was verified (*OsGS1*, accession no. AK068792; *OsGS2*, EU267952; *OsGS3*, LC385880). Alignment of the proteins encoded by the three genes is shown in Figure S3. The deduced amino acid sequences of OsGS1, OsGS2, and OsGS3 showed 62.1%, 56.8%, and 67.0% similarities, respectively, with the AtGS protein (AJ243813), and 82.7%, 61.8%, and 77.9% similarities, respectively, with the TaGS1 protein from wheat (AJ579380). We constructed a phylogenetic tree using the open reading frame (ORF) sequences of *GS* and *hGS* genes from different species of higher plants (Figure 1). *OsGS1* and *OsGS3* were positioned in the cluster to which other Poaceae *GS* genes, such as *TaGS1* and *ZmGS*, belonged, whereas *OsGS2* was separate from that cluster. Transcript sequences with high similarities to *OsGS2* were also found in wheat and barley, but the enzymatic activity of the proteins encoded has not been examined.

According to two independent prediction programs of protein subcellular localization, TargetP (Emanuelsson, Nielsen, Brunak, & Hejine, 2000) and WoLF PSORT (Horton et al., 2007), OsGS1 and OsGS2 were predicted to be chloroplastic localization. OsGS3 had a shorter amino acid sequence than other two OsGS proteins owing to 60 aa deletion in the N‐terminus (Figure S3), and it was predicted to be targeted to the cytosol or chloroplast. These localizations are the same as for AtGS, which is encoded by a single gene and two transcriptional variants encode the plastidic and cytosolic proteins (Wachter, Wolf, Steininger, Bogs, & Rausch, 2005).

**Figure 2:**
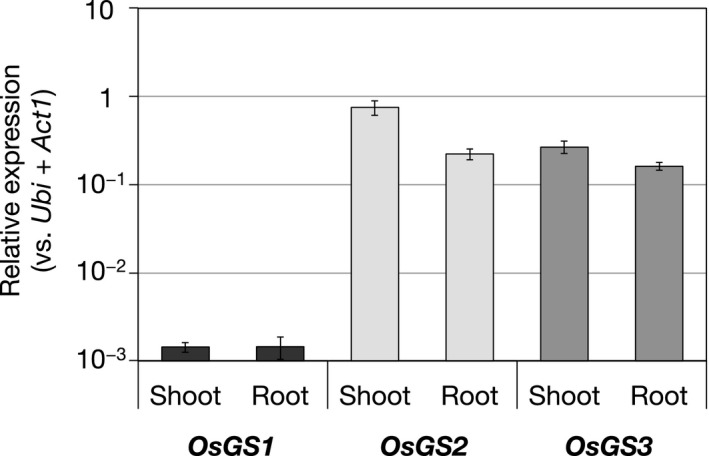
Relative expression levels of OsGS genes in the shoots and roots. Rice seedlings were hydroponically grown for 10 days under control conditions. The expression level was analyzed by RT‐qPCR. *Ubiquitin* and *Actin1* were used as internal standards for normalization. Values are expressed as means ± *SE* (*n* = 4)

The expression levels of the *OsGS* genes in shoot or root tissues were analyzed by RT‐qPCR (Figure 2). The relative expression level of *OsGS1* was much lower than those of other two *OsGS*s. The expression levels of *OsGS2* and *OsGS3* in the shoot were higher than those in the root.

### 3.2 GSH and hmGSH concentrations in OsGS RNAi transgenic plants

**Figure 3:**
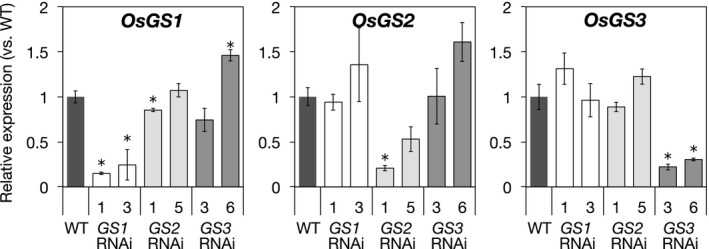
Suppression of OsGS expression in RNAi plants. WT (cv. Nipponbare) and RNAi T_1_ plants were grown hydroponically for 18 days in the control culture solution. Culture solution was renewed every 6 days. Total RNA was extracted from the shoots of individual plants. Expression levels were analyzed by RT‐qPCR. *Ubiquitin* was used as the internal standard. Expression level is presented relative to that in the WT plants. Values are expressed as means ± *SE* (*n* = 3). An asterisk (*) shows a significant difference from the WT plants (*p *<* *0.05, Dunnett test)

In order to identify the differences in physiological functions of the three GS isozymes in rice, we generated transgenic knockdown plants in which the expression of *OsGS1*,* OsGS2*, or *OsGS3* was specifically suppressed via RNA interference (RNAi). The levels of suppression of the target gene in two independent RNAi lines are shown in Figure 3. The expression level of *OsGS1* in the *OsGS1* RNAi plants was reduced to 15%–24% of that in the wild type (WT), but the expressions of the other, non‐target *OsGS*s, were not significantly suppressed. Similar specific gene suppressions were also confirmed in the *OsGS2* and *OsGS3* RNAi plants, respectively (Figure 3).

**Figure 4:**
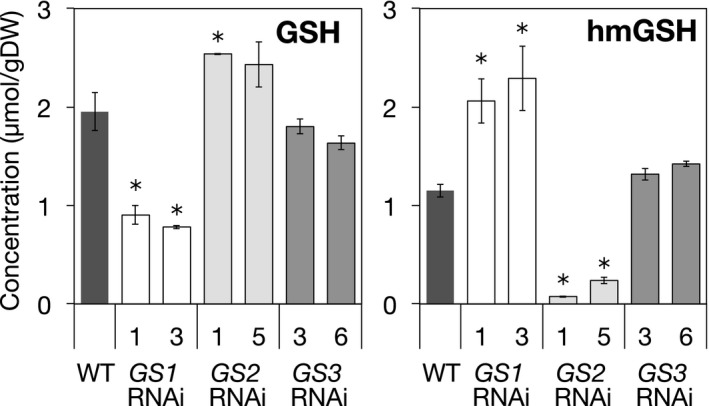
GSH and hmGSH concentrations in OsGS RNAi plants. The 10‐day‐old seedlings of RNAi plants and WT (cv. Nipponbare) plants were transferred to a culture solution supplemented with 10 μmol/L CdSO_4_. Culture solution was renewed at 5 days after transfer. Plants were harvested at 10 days after transfer, and the concentrations of thiol peptides in the shoots were determined. Values are expressed as means ± *SE* (*n* = 3). An asterisk (*) shows a significant difference from the WT plants (*p *<* *0.05, Dunnett test)

The concentrations of GSH and hmGSH in the shoots of the transgenic RNAi plants were determined. The plants were grown in the presence of a low concentration of Cd at which plant growth was not affected but that stimulated GSH production. In the shoots, GSH and hmGSH accounted for the majority of the thiol peptides. *OsGS1* knockdown decreased GSH concentration to half of that in the WT, whereas hmGSH concentration exhibited a corresponding increase in *OsGS1* RNAi plants (Figure 4). In contrast, *OsGS2* knockdown resulted in a markedly decreased hmGSH concentration and an increased concentration of GSH, compared with those in the WT (Figure 4). Suppression of *OsGS3* expression had a smaller effect on the thiol peptides than did those of the other *OsGS*s; a slight decrease in GSH and a slight increase in hmGSH were observed in the *OsGS3* RNAi plants, although those differences were not deemed to be significant following statistical analysis.

### 3.3 Enzymatic characteristic of OsGS proteins

The RNAi experiment suggests that OsGS2 is involved in hmGSH synthesis in rice. In order to investigate the enzymatic activities of OsGSs, we prepared maltose‐binding protein (MBP)‐fused OsGS proteins. The recombinant proteins were expressed in *Escherichia coli* and were purified by affinity chromatography. Purification of the proteins of interest (MBP‐OsGS1, 2, and 3) was confirmed by SDS‐PAGE (Figure S2).

**Figure 5:**
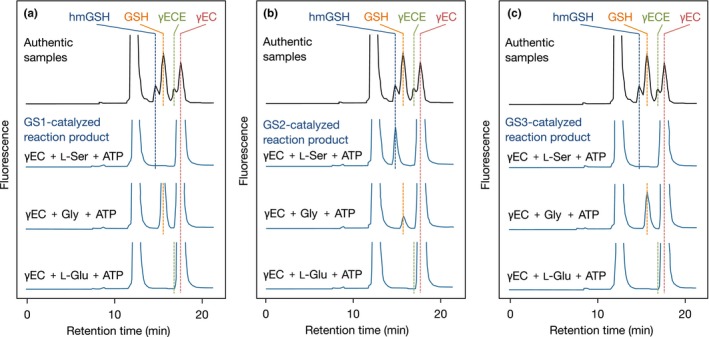
Substrate specificity of OsGS proteins. HPLC chromatogram of reaction products catalyzed by (a) OsGS1, (b) OsGS2, or (c) OsGS3. Purified MBP‐OsGS1, MBP‐OsGS2 (10 μg/ml), or MBP‐OsGS3 (0.5 μg/ml) were incubated at 30°C for 30 min in the reaction mixture containing 100 mmol/L Tris‐HCl (pH 8.0), 50 mmo/L KCl, 1 mmol/L DTT, 0.1 mg/ml BSA, 20 mmol/L MgCl_2_, 2 mmol/L ATP, 1 mmol/L γEC, and 4 mmol/L amino acid (L‐Ser, Gly, or L‐Glu). The reaction was stopped by the addition of TFA at a final concentration of 1% (v/v). Reaction products were derivatized with monobromobimane and analyzed by HPLC with fluorescence detection. Authentic reagents GSH, hmGSH, γEC, and γECE were used to identify the peaks of each thiol peptide

First, we investigated the GS activity of MBP‐OsGS1, 2, and 3. All the three proteins could catalyze GSH synthesis from γEC and Gly in the presence of ATP (Figure 5).

**Figure 6:**
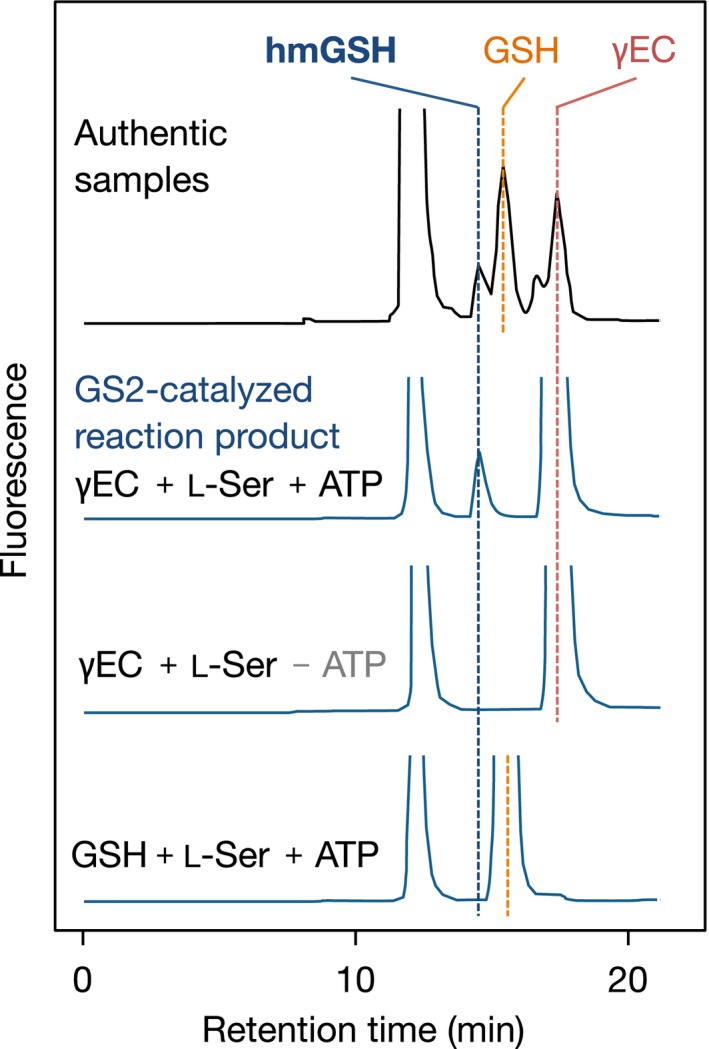
OsGS2‐catalyzed hmGSH synthesis in an ATP‐dependent manner. HPLC chromatogram of OsGS2‐catalyzed reaction product analysis. Purified MBP‐OsGS2 (10 μg/ml) was incubated at 30°C for 30 min in the reaction mixture containing 100 mmol/L Tris‐HCl (pH 8.0), 50 mmol/L KCl, 1 mmol/L DTT, 0.1 mg/ml BSA, 20 mmol/L MgCl_2_, 2 mmol/L ATP, 1 mmol/L γEC (or GSH), and 4 mmol/L L‐Ser. The reaction was stopped by the addition of TFA at a final concentration of 1% (v/v). Reaction products were derivatized with monobromobimane and analyzed by HPLC with fluorescence detection. Authentic reagents GSH, hmGSH, and γEC were used to identify the peaks of each thiol peptide

Then, we examined whether or not MBP‐OsGS2 could catalyze hmGSH synthesis, and, if so, what the substrate was for that reaction. L‐Ser and γEC or GSH as the putative substrates were incubated with MBP‐OsGS2 in the presence of ATP, and the reaction product was analyzed by HPLC. When γEC and L‐Ser were supplied in the reaction mixture containing MBP‐OsGS2, the formation of hmGSH was detected (Figures 5b and 6) in a time‐ and dose‐dependent manner (Figure S4). Heat‐inactivated MBP‐OsGS2 protein (95°C, 2 min) and the vector control protein (purified MBP) could not generate such a product. In addition, there was no product from γEC and L‐Ser in the absence of ATP, nor from GSH and L‐Ser as substrates even in the presence of ATP (Figure 6). These results indicated that OsGS2 catalyzes an ATP‐dependent hmGSH synthesis reaction from γEC and L‐Ser as co‐substrates.

On the other hand, MBP‐OsGS1 and ‐OsGS3 could not catalyze the synthesis of hmGSH (Figure 5a,c). Besides, all the three proteins could not synthesize γECE from γEC and L‐Glu (Figure 5). These results suggest that OsGS1 and OsGS3 specifically accept Gly as the co‐substrate with γEC.

**Figure 7:**
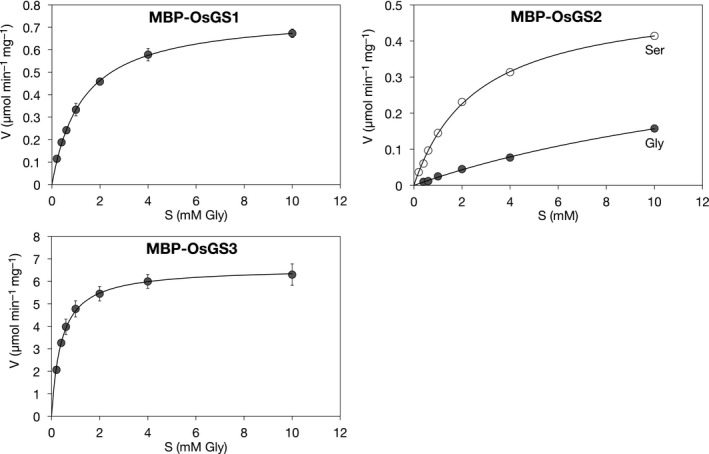
Michaelis‐Menten saturation curves of OsGS proteins for L‐Ser and Gly. Purified MBP‐OsGS1, MBP‐OsGS2 (10 μg/ml), or MBP‐OsGS3 (0.5 μg/ml) were incubated at 30°C for 5–20 min in the reaction mixture containing 0.2–10 mmol/L L‐Ser or Gly. The reaction was stopped by the addition of TFA at a final concentration of 1% (v/v). Reaction products were quantified by HPLC with fluorescence detection. Kinetic parameters were calculated by non‐linear regression analysis, using R software to fit the data to curve‐of‐best‐fit for the Michaelis‐Menten equation, *v* = *V*_max_ [*S*]/(*K*_m_ + [*S*]). Values are expressed as means ± *SE* (*n* = 3)

To determine the substrate preferences of OsGS2, we calculated apparent kinetic parameters for L‐Ser and Gly at the fixed concentration of 1 mmol/L γEC (Table 1). In the concentration range from 0.2 to 10 mmol/L L‐Ser or Gly, MBP‐OsGS2 showed a much higher initial velocity for hmGSH synthesis than for GSH synthesis (Figure 7). The *K*_m_ value for L‐Ser (2.64 ± 0.05 mmol/L) was one‐seventh that for Gly (19.3 ± 1.1 mmol/L), demonstrating that OsGS2 has a greater affinity for L‐Ser rather than for Gly.

**Table 1:** Kinetic parameters of recombinant OsGS proteins

	Substrate	*K*_m_ (mmol/L)	*V*_max_ (μmol min^−1 ^mg^−1^)
MBP‐OsGS1	Gly	1.28 ± 0.10	0.759 ± 0.015
MBP‐OsGS2	L‐Ser	2.64 ± 0.05	0.524 ± 0.005
	Gly	19.3 ± 1.1	0.459 ± 0.009
MBP‐OsGS3	Gly	0.408 ± 0.005	6.60 ± 0.41

Kinetic parameters were calculated using R software to fit the data to Michaelis‐Menten equation, *v* = *V*_max_[*S*]/(*K*_m_ + [*S*]).

Values are expressed as means ± *SE* (*n* = 3).

The kinetic parameters of MBP‐OsGS1 and ‐OsGS3 for GSH synthesis were also calculated (Figure 7 and Table 1). The *K*_m_ value of MBP‐OsGS1 for Gly (1.28 ± 0.10 mmol/L) was comparable to that of AtGS (1.51 ± 0.09 mmol/L) reported previously (Jez & Cahoon, 2004). The *K*_m_ value of MBP‐OsGS3 for Gly (0.408 ± 0.005 mmol/L), however, was lower, and the *V*_max_ value was much higher than those of the other two OsGS enzymes.

## 4 DISCUSSION

RNAi‐mediated knockdown of *OsGS2* dramatically reduced the hmGSH concentration in transgenic plants but increased the GSH concentration (Figure 4). This finding suggests that OsGS2 is involved in hmGSH synthesis and either that GSH and hmGSH are synthesized from the same substrate, γEC, or that GSH itself is a substrate for hmGSH synthesis. In vitro enzyme assays, using recombinant proteins, demonstrated that OsGS2 could directly catalyze hmGSH synthesis from γEC and L‐Ser in an ATP‐dependent manner (Figure 6). OsGS2 could also utilize Gly as the cosubstrate with γEC in vitro, but the *K*_m_ value for Gly (19.3 ± 1.1 mmol/L) was much higher than that for L‐Ser (2.64 ± 0.05 mmol/L). Considering that the concentration of free Gly and L‐Ser in the leaf tissue of monocots, such as maize and barley, is in the range 1–10 mmol/L (Chapman & Leech, 1979; Dietz, Jäger, Kaiser, & Martinoia, 1990), OsGS2 could selectively utilize L‐Ser at a physiological concentration to catalyze hmGSH synthesis.

*OsGS2*‐like *hmGS* genes may be commonly present in some species of the Poaceae. Previous reports had indicated that wheat recombinant GS proteins, TaGS1 and TaGS2, could not catalyze hmGSH synthesis from γEC and L‐Ser (Skipsey et al., 2005), even though a high concentration of hmGSH can be found in the leaves of wheat (Klapheck et al., 1992). Skipsey et al. (2005) suggested that hmGSH may arise as a result of postsynthetic modification of GSH. However, through the BLAST search, we found other putative *hmGS* transcripts in wheat and barley, which were highly homologous to *OsGS2*, and designated them as *TaGS4* and *HvGS2* (Figure 1). If the proteins encoded could be shown to catalyze hmGSH synthesis, the identity of the specific amino acid residue determining the substrate selectivity of these GS‐like enzymes may be clarified.

Enzymatic analysis of OsGS proteins revealed that OsGS1 and OsGS3 were the GSH synthetase specific for Gly. In comparison with OsGS3, the *V*_max_ value of OsGS1 was small (Table 1). Furthermore, the expression level of *OsGS1* in rice plants was lower than that of *OsGS3* (Figure 2). Nevertheless, the knockdown of *OsGS1* caused a marked decrease in GSH concentration in plants, while *OsGS3* RNAi plants did not show any significant decrease in GSH concentration (Figure 4). Distinct predicted subcellular localizations of OsGS1 and OsGS3 might explain these apparently contrasting observations. OsGS1 was predicted to be plastidic‐localized, but OsGS3 was predicted to be cytosolic or plastidic‐localized. The majority of GSH in rice plants might be synthesized in the plastids. Similar results have been obtained in other plant species. In *Arabidopsis*, γECS (GSH1) is localized exclusively in the plastids, so that γEC, a precursor of GSH, is synthesized in the plastids. As for the two transcript variants of *AtGS* (*GSH2*), the transcript variant encoding cytosolic protein is more abundant than the plastid‐targeting one (Wachter et al., 2005). However, the loss of function of CRT‐LIKE TRANSPORTER (CLT) family that mediates transport of γEC and/or GSH from plastids to the cytosol only decreases cytosolic GSH levels but does not affect the total GSH levels in *Arabidopsis* leaves (Maughan et al., 2010). It indicates that the majority of GSH is synthesized in the plastids and then it is exported to the cytosol.

Knockdown of *OsGS1* decreased GSH but increased hmGSH in transgenic plants, and knockdown of *OsGS2*, hmGSH synthetase, decreased hmGSH but increased GSH concentration (Figure 4). It can be explained by a competition of OsGS1 and OsGS2 for the common substrate, γEC, because both of them were predicted to be localized in the chloroplast. In other words, it suggests a trade‐off relation between GSH and hmGSH synthesis in rice plants.

The in vitro enzyme assay experiment showed that none of the OsGSs could catalyze γECE synthesis from γEC and L‐Glu (Figure 5), although γECE is found in rice plants at much lower concentration than those of GSH and hmGSH. Transgenic *OsGS* RNAi plants also did not exhibit reduced γECE concentrations (data not shown). These results suggest that γECE may be synthesized in rice via other pathways that are independent of GS activity. We previously reported that knockdown of the phytochelatin synthase (PCS) gene (*OsPCS2*) caused a marked decrease in γECE concentration in rice plants under Cd stress (Yamazaki, Ueda, Mukai, Ochiai, & Matoh, 2018). In maize, γECE could be detected only after the appearance of the Cd‐induced (γGlu‐Cys)_n_‐Gly phytochelatin (Meuwly, Thibault, Schwan, & Rauser, 1995). These findings suggest that γECE may be formed by transpeptidation of GSH, catalyzed by PCS, or by cleavage of the γ‐glutamyl bond of PCs in rice and maize.

We are now carrying out experiments to investigate the physiological function of hmGSH, especially with regard to alleviation of heavy metal damage in rice plants, employing *OsGSs* RNAi plants.

## 5 CONCLUSION

This study identified a rice GS‐like protein, OsGS2, as hydroxymethyl‐glutathione synthetase, which selectively utilized L‐Ser as a cosubstrate with γEC. Further investigations of OsGSs could be conductive to a better understanding of the physiological functions of GSH and hmGSH in rice.

## CONFLICT OF INTEREST

The authors declare no conflict of interest associated with the work described in this manuscript.

## AUTHOR CONTRIBUTIONS

S.Y. performed most of the experiments and drafted the article with contributions from all authors; K.O. and T.M. designed the research and supervised the experiments.
