# Supplementary material for: Rice plants have three homologs of glutathione synthetase genes, one of which, OsGS2, codes for hydroxymethyl‐glutathione synthetase
Source: Plant Direct. 2019 Feb 18;3(2):e00119. doi: 10.1002/pld3.119 (PMC6508825; doi:10.1002/pld3.119)
Supplement: Supplementary file 1 [file PLD3-3-e00119-s001.pdf]

```
CLUSTAL O(1.2.4) multiple sequence alignment

GS2 trigger sequence

OsGS2      ---------------------------------CACCAACTCCGA-TCGAACAGCCCAAT      26
OsGS1      --------------CCACGGGACAAATTGCAGAGACCACCTCGGAATTCTCCAAATTCAT      46
OsGS3      CGCAGCCCCCTCCACTTTCCTCGAAATTGCAACTCCCACCTCAAC-TATAAACTTCGGTT      59
                                              *** ***    *            *

OsGS2      CGACCATGGCCGCCGCCGCCGCCCGTTCCGGCGCGCATCCGCCATCGTTCGCCTCCTCGC      86
OsGS1      CGCCAATGTCCTCCTACGTCACCACCCC-------CCACCACCACCACCACCATGGCTGC      99
OsGS3      CGACAGTTCGGCACT-CGACA---ATCT--------CGCAGAGAGAGTGAAGCTGAGATT      107
           ** *  *      *  ** *                  *    *         *

OsGS2      TCGGCCGGTGCAGGGTTCTTCCAGTCGTGGTGGTGCGTCGGCCGGGCGGTGCCGCGAGGC      146
OsGS1      TGCTCCGGCAGCCGCCGCCTCCAGGCACCGGC----GCCTCCCGCCCGCCCCCGCCTCGT      155
OsGS3      GCAATTTGCAAATATCCCCTCGAGAAATCATAATTTGTTTTTCA----------------      151
                  *         * ** **            *     *

OsGS2      CGTCACCGCTGCTCGCTCCGGCGAGGTGCGCCGCCGCCGTCGG--GACGGCGGCGCCCAA      204
OsGS1      CGTCGCCGCCGCC---GCGAGACACGTGGCCCTCCCCCCCCGCCGCGCGGTGGCCTCGAG      212
OsGS3      --------CTC-----CCTAATCCCCCTCCAGTC-------------------CACCGCC      179
                   *        *               *                   *  *

OsGS2      GGTGGAGGGCGGCGG---ACGGCGGTCGTCGGAGCAGGGGCAGCTGGCGGTGGCGCCGGC      261
OsGS1      GGCGATGAGCGCCGAGGCGCCCCTGGGTGTCGCGCCGGCGGCGGCGGAAGAGGAGATGGC      272
OsGS3      CGCGATGAGCGCCGCCGCGGAGGGGAGGCCGCCGGCGGCGGCGGCGGCGGGTGAGATGGT      239
            * *  * *** **          *        *  ** *  *  **  *  * *  **

OsGS2      GCGGCTGGTGGATGAGCTGGTCGAGGAG    289
OsGS1      GGCGGTGGTGGACGAGATGGCGGAGGAG    300
OsGS3      GCGG------------------------    243
           *  *


GS1 and GS3 trigger sequences

OsGS1      GTCACTCACCATGCACCATCACACCAAATA-TCAAATCAATCTTGATAAGCATATGCTCC      59
OsGS3      ----ACCGACAAGGCGATGTAGACATGATTGTTGAACATTTTCT------CAGAT---TT      47
OsGS2      ---ACATGACATGGTAAGACATGCGAAACATGTGTATCGGTTTG------AATAT---GG      48
                    ** *       *  *   *       *    *          * **

OsGS1      AGTCAACTTCAACCAGTTTCTGAGGCATTCCTGTCACA-CCGTAGAATGAA---------      109
OsGS3      AGCCTGCTCATTTCTGATGGACAGCAAATTCCAAAACAAGAACACATTGTATCGTGTTTC      107
OsGS2      ATAATTCTCCAAGCAGGTTATCAGAGAATTCAT----------AATTTGTATGCTATTA-      97
           *     **     * * *    **  * * *            *   ** *

OsGS1      --------TAGACCAAAAAAAATCTGAAATATTCTTCTCTAGTTTCTTCTTCTCTTCTGA      161
OsGS3      AAGTGTACTATATGCTTGAAGCTGAGGAGCATTATAGGCTTATAGC---TTCTCATCTGA      164
OsGS2      -------CTTTTAGCAGGAATCATACCAATGTTATT-------TCC---ATGACACACTT      140
                   *         **       *   ** *          *    *  *

OsGS1      AATTTTGAATTGAATCTGTCTTTTGTGATGTCATTCTTGCAGTGAGGAAGCAAGGTAGCA      221
OsGS3      TTATTTCC--------------TTGGGACGTTATGATAATTATCCCTATTTATGCTCCCG      210
OsGS2      TTTTTTAT--------------TTGGCATGATTTCGCTATCAGCCGAGATTGAGGTACCA      186
              ***                ***  * *   *                   * *  *

OsGS1      AGAGTTACAATTGAC--------AGCTAGCAGACACTGGAATGAACCTGGCTGACAGAAG      273
OsGS3      ATATGTAATTTTGTACTACAAGGTGGTGTCTTCAGTTGGCAGTTTACTTTAGATGTG---      267
OsGS2      TTATTCCCATGTATA--AAAATGTTATAGCTA----TCATATTTTAATGTATGAGTA---      237
             *        *              *  *      *   *      *

OsGS1      GTTGCAATCTCAGGATTCGGTTGAGCACCAGTT        306
OsGS3      ----GTGTTTGCAGTTTTTGAATGGCA------        290
OsGS2      --T-CAATTTGCTTATATTGT------------        255
                  * *     *   *
```

Supplemental Figure S1. Alignment of RNAi trigger sequence regions of *OsGS*s.

Alignment was conducted by Clustal Omega program.

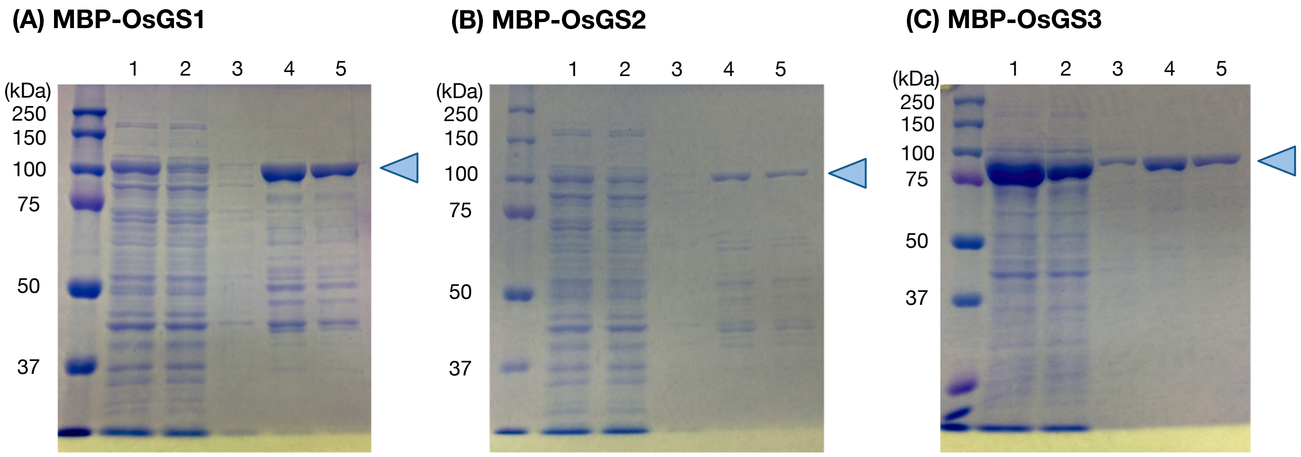

Supplemental Figure S2. SDS-PAGE of fractions following the purification of MBP-OsGS proteins. MBP-OsGS1 (A), MBP-OsGS2 (B), and MBP-OsGS3 (C). SDS samples were stained for total proteins using CBB. Blue triangle shows purified MBP-OsGS1 (MW: 102.4 kDa), MBP-OsGS2 (MW: 101.5 kDa), and MBP-OsGS3 (MW: 95.6 kDa). Lane 1: crude extract. Lane 2: flow-through. Lane 3: wash. Lane 4: purified MBP-OsGS protein #1 eluted from resin (OsGS3, 10-fold diluted). Lane 5: purified MBP-OsGS protein #2 eluted from resin (OsGS3, 10-fold diluted).

```
OsGS1     1 MSSYVTTPHHHHHHGCCSGSRRLQAPAPPARPRLVVAAAARHVALPPRRAVASRAMSAEA
OsGS2     1 MAAAAARSGAHPPSFASSLGRCRVLPVVVVR-RPGGAARPSPLLAPARCAAAVGTAAPKV
OsGS3     1 ---------------------------------MSAAAEGR----PP-------------

OsGS1    61 PLGVAPAAAEE----EMAAVVDEMAEEAAVWCAVHGLVVGDRAEPRSGTIPGVGLVHAPF
OsGS2    60 EGGGRRSSEQGQLAVAPARLVDELVEEALVWSSQHGLVVGDKNHPRSGKAPGVGLLHAPF
OsGS3    11 ----------------AAAAAGEMVREATAWCALHGLVVGDRADPRSGTVPGVGLVHAPF

OsGS1   117 ALLPTRFPASFWKQARELAPIFNDLVDRVSLDGEFLQDSLSRTRQVDDFTSRLLDIHAKM
OsGS2   120 ALLPMSFSKVYWDQAVELAPLFNELVDRVSLDGDFLQETLARTKEVDSFTGRLLDIHAKM
OsGS3    55 SLLPTHLPESHWRQACELAPIFNELVDRVSLDGDFLQDSLSKTKQVDDFTSRLLEIHRKM

OsGS1   177 MEVNKEEDIRLGLHRSDYMLDSGTNSLLQIELNTISSSFPGLSSLVSELHRTLLNRHGKV
OsGS2   180 MKLNKKEDVRLGLTRSDYMIDGATDQLLQVELNTISTSSNGLACGVCELHRNLIRQHERE
OsGS3   115 MEINKEENIRLGLHRSDYMLDSETNSLLQIELNTISASFPGLGSLVSELHRTLIDQYGHL

OsGS1   237 LGLDSKRIPQNWAATQFAEALSMAWTEFNNKSAVIMMVVQPEERNMYDQYWLINHLKESH
OsGS2   240 LGLDPESVVGNTAIAQHAEALAGAWAEFNNQSSVVLVVVQPEERYMYDQYWITVALREMY
OsGS3   175 FCLDSKRVPGNEASSQFAKALARAWDEFNVDSAVIMMIVQPEERNMYDQYWLAKHLKESH

OsGS1   297 GVKTIRKTLAQVEAEGQVLPDGTLVVDGQTVSVVYFRAGYSPNDYPSEAEWRARLLMEQS
OsGS2   300 GVTTIRKTMAAIDAEGELRPDGTLTIDGLPVAVVYFRAGYTPNDYPSEAEWRARLLIECS
OsGS3   235 GITTIRKTLSEVEAEGHVLPDGTLVIDGKTVSVVYFRAGYTPNDYPSEAEWAARLLLEQS

OsGS1   357 SAIKCPSISYHLVGTKKIQQELAKPNILERFLNNKEDIAKLRKCFAGLWSLDNEEIVKTA
OsGS2   360 SAIKCPSIAHHLVGTKKIQQELAKENVLERFLDNKADIEKVRKCFAGLWSLENDSIVMSA
OsGS3   295 SAVKCPSISYHLVGTKKIQQELARPNVLERFLENKEEITKIRKCFAGLWSLDDEEIVKSA

OsGS1   417 IEKPDLFVLKPQREGGGNNIYGYDLRETLVRLQKEQGEALAAYILMQRIFPRASLTHLVQ
OsGS2   420 IESPELFVLKPQREGGGNNIYGDNLRETLISLKKDGSNELAAYILMQRIFPPASLCYLVR
OsGS3   355 IQKPELFVLKPQREGGGNNIYGIDVRETLIRLQKEGGDALAAYILMQRIFPKASLSNLVR

OsGS1   477 GGVCFEDLTISELGIFGAYLRNKDKVVLNNQCGYLMRTKVSSSNEGGVAAGFAVLDSILL
OsGS2   480 DGTCIRENAVSEFGIFGAYLRNKDRVIINDQCGYLMRTKAASLNEGGVVAGYAFLNSVFL
OsGS3   415 GGVCHEALTISELGIYGAYLRNNDKVVMNEQSGYLMRTKVSSSDEGGVAAGFAVLDSLYL

OsGS1   537 TDEW-
OsGS2   540 T----
OsGS3   475 TDKAM
```

Supplemental Figure S3. Alignment of three OsGS proteins.

Alignment was conducted by ClustalW 2.1 program in the DNA Data Bank of Japan (DDBJ).

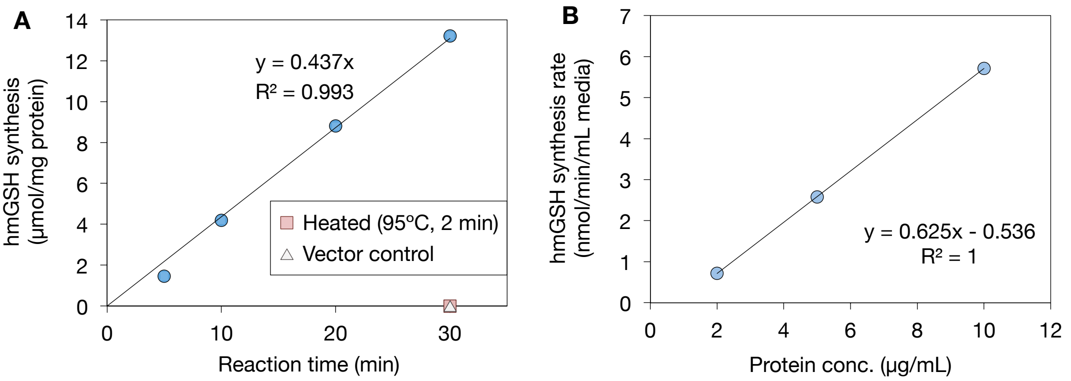

Supplemental Figure S4. OsGS2-catalyzed hmGSH synthesis in a time- and dose-dependent manner. (A) Time-dependent hmGSH synthesis. Purified MBP-OsGS2 (10 µg mL^{-1}) was incubated at 30ºC for 5–30 min in the reaction mixture containing 100 mmol L^{-1} Tris-HCl (pH 8.0), 50 mmol L^{-1} KCl, 1 mmol L^{-1} DTT, 0.1 mg mL^{-1} BSA, 20 mmol L^{-1} $MgCl_2$, 2 mmol L^{-1} ATP, 1 mmol L^{-1} γEC, and 4 mmol L^{-1} L-Ser. Heat-inactivated MBP-OsGS2 protein (95ºC, 2 min) and vector control protein (MBP) were used as a negative control. (B) Protein dose-dependent hmGSH synthesis. Purified MBP-OsGS2 (2, 5, or 10 µg mL^{-1}) was incubated at 30ºC for 5–20 min in the reaction mixture. The reaction was stopped by the addition of TFA at a final concentration of 1% (v/v). Reaction products were derivatized with monobromobimane and analyzed by HPLC with fluorescence detection. Thiol peptides were quantified by reference to the GSH standard.

Supplemental Table S1. The sequences of primers used in this study.

GS1.RNAi.F	5′-CACCGTCACTCACCATGCACCATC-3′
GS1.RNAi.R	5′-AACTGGTGCTCAACCGAATC-3′
GS2.RNAi.F	5′-CACCAACTCCGATCGAACA-3′
GS2.RNAi.R	5′-CTCCTCGACCAGCTCATCC-3′
GS3.RNAi.F	5′-CACCGACAAGGCGATGTAGAC-3′
GS3.RNAi.R	5′-TGCCATTCAAAAACTGCAAA-3′
Gus-linker.R	5′-ATCCACGCCGTATTCGG-3′
Ubiquitin. F	5′-AGAAGGAGTCCACCCTCCACC-3′
Ubiquitin. R	5′-GCATCCAGCACAGTAAAACACG-3′
Actin1. F	5′-ATCCTTGTATGCTAGCGGTCGA-3′
Actin1. R	5′-ATCCAACCGGAGGATAGCATG-3′
GS1.ex-F	5′-GCGCACCAAAGTTTCTTCATC-3′
GS1.ex-R	5′-GACCCTCTGCTTTACCACTCATC-3′
GS2.ex-F	5′-TGGGCTGAGTTCAACAATCAA-3′
GS2.ex-R	5′-CACCCCATACATTTCTCTCAAGG-3′
GS3.ex-F	5′-TTGAAGCCCCAACGTGAA-3′
GS3.ex-R	5′-GGCAAACACCACCACGAA-3′
GS1.proF	5′-ATGTCCTCCTACGTCACCACC-3′
GS1.proR	5′-ACTGCCTGCAGGTTACCACTCATCTGTGAGGAG-3′
GS2.proF	5′-ATGGCCGCCGCCGCCGCCCGTTC-3′
GS2.proR	5′-GCTCCCTGCAGGTCATGTCAGGAATACGCTGTTC-3′
GS3.proF	5′-ATGAGCGCCGCCGCGGAGGGGAG-3′
GS3.proR	5′-ACTACCTGCAGGCTACATCGCCTTGTCGGTTAAG-3′
GS1.seqR	5′-TCCGTAGCTTGGCAATGTC -3′
GS2.seqR	5′-GTACCTCAATCTCGGCTGATAG-3′
GS3.seqF	5′-ACTTCGGTTCGACAGTTCGGCAC-3′

Supplemental Table S2. Percentage identity matrix of RNAi trigger sequence regions.

	OsGS2	OsGS1	OsGS3
GS2 trigger	-	53.82%	39.61%
	**OsGS1**	**OsGS3**	**OsGS2**
GS1 trigger	-	41.83%	44.35%
GS3 trigger	-	-	48.22%

Alignment was conducted by Clustal Omega program.
