# Supplementary material for: Rice plants have three homologs of glutathione synthetase genes, one of which, OsGS2, codes for hydroxymethyl‐glutathione synthetase
Source: Plant Direct. 2019 Feb 18;3(2):e00119. doi: 10.1002/pld3.119 (PMC6508825; doi:10.1002/pld3.119)
Supplement: Supplementary file 2 [file PLD3-3-e00119-s002.pdf]

Response to reviewers' comments

We are grateful for your careful reading and insightful comments to our manuscript. We revised our manuscript in accordance with your comments, and all the changes made on the manuscript are highlighted in yellow. Please see below our responses to each of your comments.

Revision on the comments from Reviewer #1

1. **Page2 Abstract L2: "other tripeptides" in here should be "glutathione-like other tripeptides". A mere "tripeptides" corresponds to various kinds of tripeptides.**

Thank you for your comment. We revised it.

2. **Page4 L9: "we have tried to identify" should be just "we identified". "tried to~" gives negative impression like "tried to~ ... but failed".**

Thank you for your advice. We revised it.

3. **Page 5 L1~4: Both of mol L-1 and g L-1 are used for concentrations of chemicals in the culture solution. One of them, mol -1 or g L-1, should be used consistently for all of chemicals.**

We revised it to use mol L^{-1} consistently.

4. **Page9 L13: Explain why MBP-OsGS3 was used 0.5 microg mL-1 although other two proteins are used at 10 microg mL-1.**

We adjusted the concentration of proteins to keep the linearity of GSH synthesis with time up to 20 minutes. MBP-OsGS3 was used at the lower concentration because it had a higher specific activity than other two OsGS proteins. We added the explanation in page9 L13.

5. **Page11 L11: "Thiol peptides were quantified by the GSH standard."**
   Explain why standards of hmGSH,gammaEC, and gammaECE were not used for quantifying hmGSH, gammaEC, and gammaECE respectively.

We found a gradual decrease of peak area for standard peptides during long-term storage of the stock solutions in a freezer. We should prepare fresh standard solutions for every time of our determinations, but it was difficult for us because the authentic reagents of GSH-related compounds were a bit expensive. Therefore, we

decided to use a freshly prepared GSH solution and peak ratios for the quantification of hmGSH, gammaEC, and gammaECE, and the authentic reagents of them were used for the determination of their retention time. We, however, used freshly prepared γEC solution as the substrate of enzymatic assays.

6. **Page13 and Supplemental Fig. S3: OsGS1 and OsGS2 have longer N-terminal sequences compared to OsGS3. Check if these Ne-terminal sequences contain any signal or target peptides to specific organelles by computer prediction and describe about it in the text.**

Thank you for your suggestion. Predicted localizations were different among them. We added descriptions on this point in page13 L11–16 and in page18.

7. **Page15 L5: "in a time- and dose-dependent manner".**
   In Fig5A does not show time or dose dependent formation of hmGSH. If there is a data showing such "time- and dose-dependent manner", show in the manuscript.

We added the data in new Supplemental Fig. S4.

8. **From Page 16-18 Discussion: In discussion, authors focused on only OsGS2. As they obtained important results also for OsGS1 and OsGS3 by RNAi lines and enzyme assays with their recombinant proteins, they should include discussion about physiological functions of OsGS1 and OsGS2, for example, which is the major enzyme contributing on GSH synthesis and possible differences in roles of these two proteins in GSH synthesis.**

Thank you for your suggestion. We added the discussion about physiological functions of OsGS1 and OsGS3 in page17–18.

9. **Figure 4: hmGSH is increased in GS1RNAi and GSH is increased in GS2 RNAi. Is it because gamma-EC, the substrate for both GSH and hmGSH synthesis is used for hmGSH synthesis in GSRNAi and for GSH synthesis in GS2 RNAi? Discuss about it.**

We discussed it in page18 L12–15.

10. **Figure 5: Explain about A and B in the legend.**

We replaced Fig. 5 and added explanations on panels.

Revision on the comments from Reviewer #2

1. **In this study, three rice glutathione synthetase (OsGS1, OsGS2, OsGS3) genes were analyzed, and authors claimed that OsGS2 functioned as a hydroxylmethyl-glutathione synthetase responsible for the biosynthesis of hydroxylmethyl-glutathione (hmGSH, Glu-Cys-Ser).**
2. **In arabidopsis, glutathione synthetase is able to couple Glycine into γ-EC both in the chloroplast and cytosol. Therefore, tissue specificity and subcellular localization are important information to examine the physiological function of GS in rice. Authors should provide more information.**

Thank you for your suggestion. Computationally predicted localizations were different among OsGS1, OsGS2, and OsGS3. We add descriptions on this point in page L11–16 and in page18.

3. **In Figure 3, RNA expression of OsGS1-RNAi, OsGS2-RNAi, OsGS3-RNAi was analyzed to present the suppression of specific GS gene in rice. How about total GS activity? Furthermore, it will be nice to see the attenuation of each specific isozyme by gel zymography analysis.**

Thank you for your comment. We had tried to determine the total GS activity in crude extracts from rice plants but found that it was easily inactivated and showed low activity. Thus, we deemed it difficult to quantitatively evaluate the total GS activity and compare the difference between WT and RNAi plants.

4. **In Figure 6, Michaelis-Menten saturation curves of OsGS2 for L-Ser and Gly demonstrated that L-Ser has a greater affinity for L-Ser rather than for Gly. But the saturation curves of OsGS1 and OsGS3 were showed in the supplementary S5, not showed Fig. 6. Since substrate specificity is an important biochemical index of an enzyme, I would suggest authors merge S5 to Fig.6. Moreover, in addition to Gly, authors should provide more data on Michaelis-Menten saturation curves of OsGS1 and OsGS3 for Ser.**

We merged previous Fig. 6 and Supplemental Fig. S5 into new Fig. 7. In addition, we merged previous Fig. 5 and Supplemental Fig. S4 into new Fig. 5. As shown in new Fig. 5, OsGS1 and OsGS3 were specific to glycine and did not show any activity to utilize Ser as a substrate. Therefore, we could not provide data on Michaelis-Menten saturation curves of OsGS1 and OsGS3 for Ser.

5. **In the quantification of thiol peptide, mBBr fluorescent dye was used for HPLC analysis. From the signal showed in the HPLC chromatography, retention and separation are not enough to clearly**

distinguish peaks of different thiols. Mass spectrum is suggested for the analysis of different thiol peptides.

Regarding hmGSH and GSH, the difference in retention times between the two peaks was about one minute in our HPLC analysis. We think it is enough for the distinction of the two peptides, and therefore we decided to use our HPLC data in this manuscript. We, however, want to try mass spectrometry analysis in the near future. Thank you for your good suggestion.

6. **OsGS2 functioned as a hydroxylmethyl-glutathione synthetase and responsible for the biosynthesis of hydroxylmethyl-glutathione (hmGSH, Glu-Cys-Ser). Will overexpression of OsGS2 increase the accumulation of hmGSH in rice?**

Yes, we suppose so. We are generating such overexpression lines. We will characterize their phenotypes in our next paper to obtain clues for the physiological role of hmGSH.

7. **Journal abbreviation was not corrected in several references:**
   P.19, Line 9: Transgenic Research
   P.19, Line 15: Methods in Enzymology
   P19. Line 17: Physiologia Plantarum
   P.20, Line 3: Botanica Acta

Thank you for pointing it out. We revised them.

Reviewer Comments:

Reviewer #1:

Comments to the Author (Required):

Authors answered all of the reviewer's comments precisely and improved their manuscript. I am satisfied with their revision.

Reviewer #2:

Comments to the Author (Required):

This manuscript has been significantly improved and most of the points of concern have been addressed.
